# The complete chloroplast genome sequence of *Huodendron tibeticum* (J.Anthony) Rehder (Styracaceae)

**DOI:** 10.1080/23802359.2020.1860695

**Published:** 2021-01-21

**Authors:** Xiaoyu Jiang, Yaoqin Zhang, Lili Tong, Xiaogang Xu, Yukun Tian, Hongchao Wang

**Affiliations:** a Co-Innovation Center for Sustainable Forestry in Southern China, College of Biology and the Environment, Key Laboratory of State Forestry and Grassland Administration on Subtropical Forest Biodiversity Conservation, Nanjing Forestry University, Nanjing, China; b State Environmental Protection Scientific Observation and Research Station for Ecology and Environment of Wuyi Mountains, Nanping, China; c School of Horticulture & Landscape Architecture, Jinling Institute of Technology, Nanjing, China

**Keywords:** *Huodendron tibeticum*, complete chloroplast genome, phylogenomics, Styracaceae

## Abstract

*Huodendron tibeticum* (J.Anthony) Rehder, which plays an important role in ecology and economy, is a deciduous species of Styracaceae. The authors sequenced, assembled, and annotated the chloroplast (cp) genome of *Huodendron tibeticum* using the sequencing data from Illumina Novaseq platform in this study. The complete cp genome of *H. tibeticum* is 159,320 bp in length, including a large single-copy (LSC) region of 87,795 bp, and a small single-copy (SSC) region of 18,989 bp. It contains 130 genes, including 37 tRNA genes, 8 rRNA genes, and 85 protein-coding genes. The overall GC content of *H. tibeticum* chloroplast genome is 36.66%. The phylogenetic analysis suggests that *H. tibeticum* is a sister species to *H. biaristatum* in Styracaceae.

*Huodendron tibeticum* (J.Anthony) Rehder is a prominent species of Styracaceae, 6–25 m tall trees or shrubs, and 25 cm diameter of trunk at breast height, which mainly distribute in dense forests at an altitude of 1000–3000 m in the west and southwest (northeast Guangxi, Guizhou, west Hunan, southeast Xizang, Yunnan) of China and Sino Indian Peninsula (Huang and Grimes 2003). It is valued for its timber, ornamental, perfume, and afforestation purposes. However, there has been little progress on its complete chloroplast genome. In this work, we characterized the complete cp genome sequence of *H. tibeticum* (GeneBank accession number: MT806103) based on Illumina pair-end sequencing data to provide a valuable complete cp genomic resource.

The fresh leaves of *H. tibeticum* were collected from Qingxiu Mountain (N 22.0780, E 108.3780) in Nanning, Guangxi, China. And total genome DNA was extracted using PlantDNA Kit (Genepioneer Biotechnologies, Nanjing, China). The herbarium of Nanjing Forestry University has deposited the voucher specimen (accession number 2020092). Using ultrasound to break DNA and the fragments of DNA were passivated, repaired, and bonded. The DNA fragments were selected by agarose gel electrophoresis. The sample of genome sequencing library was formed by PCR amplification, which was carried out on Illumina Novaseq platform by Nanjing Genepioneer Biotechnologies Inc. (Nanjing, China), and read long for PE150 sequencing.

The original reading was filtered by fastp (version 0.20.0), and the clean data were assembled into chloroplast genome using SPAdes (Bankevich et al. 2012). Next, the reference sequence (Genebank accession number: NC041127.1) was used for quality control after assembly. Finally, the assembled genome was annotated using CpGAVAS (Liu et al. 2012) and the physical map of *H. tibeticum* cp genome was drawn by the OGDRAW (Greiner et al. 2019). Based on the Maximum Likelihood (ML), the phylogenetic tree was concluded by MAFFT (Katoh et al. 2019) and MEGA (version 7) (Kumar et al. 2016).

The complete chloroplast genome sequence of *H. tibeticum* was 159,320 bp in length. The genome had a typical quadripartite structure including a pair of IR (IRa and IRb) regions of 26,268 bp that were separated by an LSC region of 87,795 bp and a SSC region of 18,989 bp. A total of 130 genes were encoded, including 8 rRNA genes (4 rRNA species), 37 tRNA genes (30 tRNA species), and 85 protein-coding genes (79 CDS species). Most of the genes occurred in a single copy; however, six protein-coding genes (*ndhB, rpl2, rpl23, rps12, rps7* and *ycf2*), seven tRNA genes (*trnA-UGC, trnI-CAU, trnI-GAU, trnL-CAA, trnN-GUU, trnR-ACG* and *trnV-GAC*), and four distinct rRNA gene (*23S, 16S, 5S* and *4.5S*) are duplicated. A total of 10 protein-coding genes (*accD, atpF, ndhA, ndhB, petB, petD, rpl16, rpl2, rpoC1, rps16*) contained 1 intron while the other 2 genes (*rsp12, ycf3*) had 2 intron each. The overall GC content of the chloroplast genome is 36.66%. In addition, the GC contents of the LSC, SSC and IR regions are 34.74%, 30.26% and 42.18%, respectively.

To reveal the phylogenetic evolution of *H. tibeticum*, we constructed a ML phylogenetic tree based on 36 cp genomes from Styracaceae and 4 cp genomes as outgroups from 2 taxa (Actinidiaceae, Symplocaceae). We found that *H. tibeticum* was clustered with other families of Styracaceae with 100% boot-strap values (Figure 1). What’s more, *H. tibeticum* was highly supported to be a sister species to *Huodendron biaristatum* in Styracaceae.

**Figure 1.**
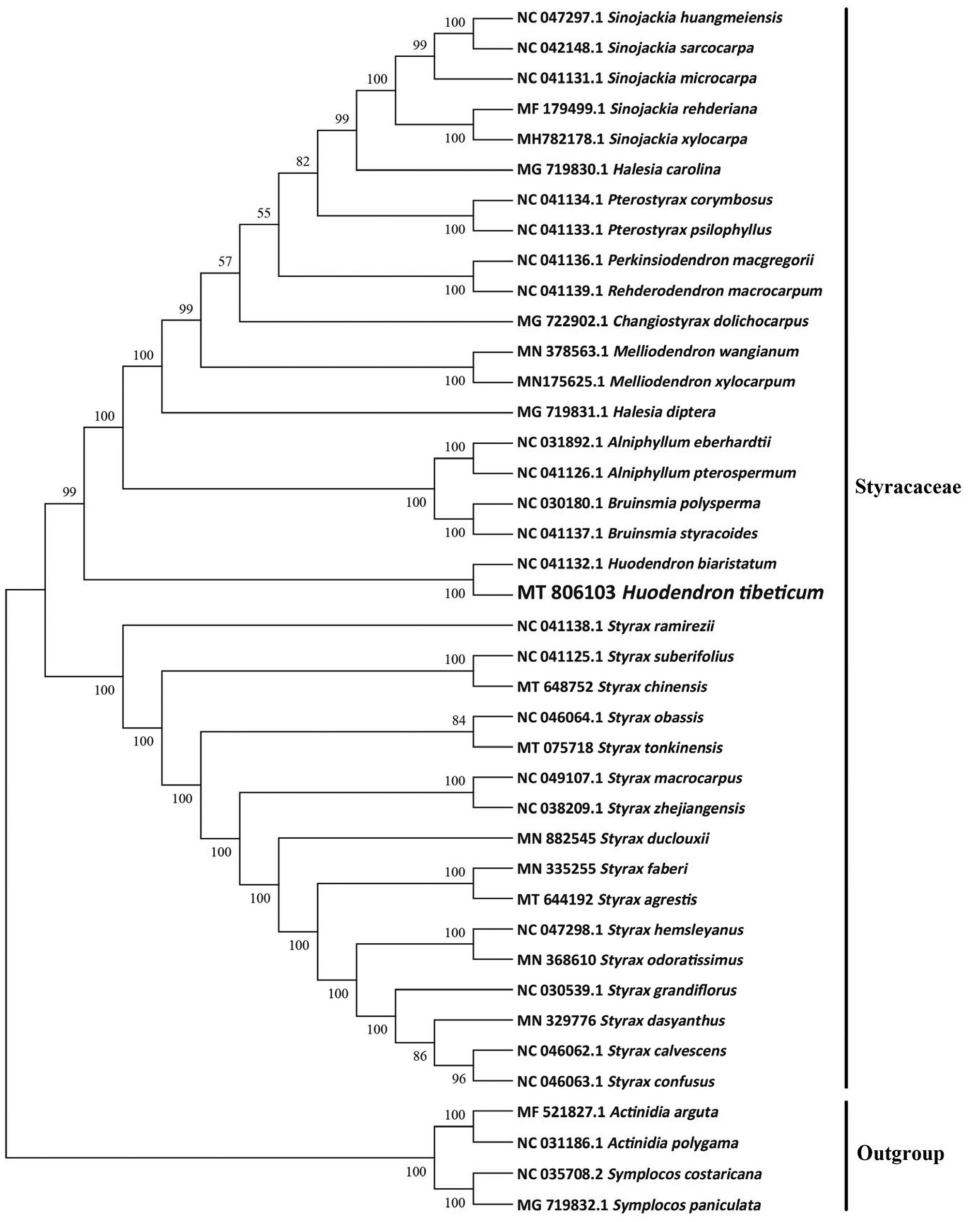
A maximum-likelihood tree was constructed based on the chloroplast genomes of 40 species. *Actinidia polygama, A. arguta, Symplocos paniculata* and *S. costaricana* were used as outgroups. The bootstrap supported the values shown at the branches.

## Data Availability

The data are accessible from https://pan.baidu.com/s/1gcgDnY3I1nzzLYpcRUiOow (password: b7na); https://pan.baidu.com/s/1IRQxW8SP8gV7PK6Spz0xrg (password:1kn4).
